# Improving communication for interdisciplinary teams working on storage of digital information in DNA

**DOI:** 10.12688/f1000research.13482.1

**Published:** 2018-01-10

**Authors:** Emily E. Hesketh, Jossy Sayir, Nick Goldman

**Affiliations:** 1 Wellcome Sanger Institute, Hinxton, CB10 1SA, UK; 2 European Molecular Biology Laboratory, European Bioinformatics Institute, Hinxton, CB10 1SD, UK

**Keywords:** DNA-storage, digital information storage in DNA, synthetic biology, glossary, communication, controlled vocabulary, short plain-language summaries, interdisciplinary collaboration

## Abstract

Close collaboration between specialists from diverse backgrounds and working in different scientific domains is an effective strategy to overcome challenges in areas that interface between biology,

chemistry, physics and engineering. Communication in such collaborations can itself be challenging.  Even when projects are successfully concluded, resulting publications — necessarily multi-authored — have the potential to be disjointed. Few, both in the field and outside, may be able to fully understand the work as a whole. This needs to be addressed to facilitate efficient working, peer review, accessibility and impact to larger audiences. We are an interdisciplinary team working in a nascent scientific area, the repurposing of DNA as a storage medium for digital information. In this note, we highlight some of the difficulties that arise from such collaborations and outline our efforts to improve communication through a glossary and a controlled vocabulary and accessibility via short plain-language summaries. We hope to stimulate early discussion within this emerging field of how our community might improve the description and presentation of our work to facilitate clear communication within and between research groups and increase accessibility to those not familiar with our respective fields — be it molecular biology, computer science, information theory or others that might become relevant in future. To enable an open and inclusive discussion we have created a glossary and controlled vocabulary as a cloud-based shared document and we invite other scientists to critique our suggestions and contribute their own ideas.

## Introduction

As we tackle increasingly complex issues throughout science, a breadth of knowledge is often necessary to devise novel solutions — something frequently achieved through interdisciplinary collaborations. The inherent diversity within interdisciplinary teams stimulates knowledge exchange, creativity or even a change in perspective; however, it can be very challenging. We work within an emerging field in synthetic biology, repurposing DNA as a storage medium for digital information. Advancing from early proof-of-principle studies in the high-throughput era ^1, 2^ (see references therein for historical perspective) towards a more reliable, refined and functional large-scale DNA storage system ^3, 4^ raises unique challenges that can only be resolved through a broad collaborative effort between biochemical and DNA sequencing specialists, computer and molecular scientists, information theorists and others. This body of research has gained considerable interest both within the research community and with the public, and this has further emphasised the need to address our communication and the presentation of our work.

### Interdisciplinary teams make significant advances in life sciences

Intersection between these fields is clearly beneficial. Information theory has already underpinned many advances in life sciences, from adapting Levenshtein coding to create error-correcting molecular barcodes used in multiplexed DNA sequencing ^5^ to Burrows-Wheeler transformation of reference genomes implemented in several short read aligners ^6– 8^. A molecular biologist may see the process of storing information in DNA as a very physical process, progressing from DNA synthesis (writing) to amplification (copying) to sequencing (reading). To an information theorist, this is a noisy channel: a series of transformations through which information is transmitted and the outputs observed. Differences in the way experts in these different fields describe their data and results can hinder collaboration and restrict impact. As a result, publications have the potential to be an ineffective hybrid of accepted nomenclature and data presentation within the intersecting fields with few readers, both in the team and outside, able to fully understand the publication as a whole.

### Unambiguous communication can be challenging and misunderstandings can pass unnoticed

Unsurprisingly, common nomenclature between the intersecting disciplines has disparate meanings. Use of the word ‘qubit’ can lead you to believe that some DNA needs quantifying ^9^ or you may be discussing quantum information or quantum field theory ^10^. This complicates communication; misunderstandings have the potential to pass unnoticed, only becoming apparent downstream. Examples of such misunderstandings are the use of the words errors, erasures, and substitutions when retrieving data through DNA sequencing. To an information theorist, an ‘error’ refers to a falsely read symbol, for example when an A in the DNA sequence is falsely read as a C, distinct from an insertion or deletion. An ‘erasure’ would be a read that was possibly so uncertain that it is neither called as an A, C, G or T, but distinct from a ‘deletion’ in that the read is not simply missed but we are made aware that there is a missing symbol at this position in the DNA string. An ‘insertion’ is a symbol read, when no symbol should exist. To a molecular biologist and DNA sequencing expert, all of these would be described as read ‘errors’. To them, errors in the information theoretic sense would be called substitutions.

## A glossary and controlled vocabulary for DNA-storage

DNA-storage has become a popular research field, with a number of interdisciplinary teams forming and collaborating in an attempt to make viable information storage systems that capitalise on DNA’s numerous advantages ^11^. To alleviate confusion and improve daily communication within and between these groups we propose, and have begun to implement, two measures: a glossary and a controlled vocabulary.

### Glossary

We have created a glossary defining basic terms in molecular biology, information theory and computer science etc. that are relevant to DNA-storage, for those unfamiliar with one or more of these disciplines. This proved to be a useful aid in early discussions within our team and helped to identify areas of nomenclature ambiguity which if not addressed may have complicated communication downstream. We have already experienced the advantages of sharing this within our team and with collaborators to facilitate exchange of ideas with them.

Our glossary is held on a cloud storage system, and can be found at https://goo.gl/x6B73Q or https://rebrand.ly/dna-storage-glossary. To allow an open and inclusive discussion of how we might improve communication within this emerging community, we encourage others to critique and contribute to the glossary. The document permits “Suggestions” (proposed edits) and “Comments” to be added, and we will review these regularly and update the document as a resource for our research community.

### Controlled vocabulary

Leading on from this, we are developing an evolving controlled vocabulary allowing team members to communicate precisely. This has been particularly beneficial during technical discussions — for instance, to us *data packet* refers to part of a DNA sequence that decodes to digital information, and excludes parts that are designed to facilitate DNA sequencing or indexing.

Use of a controlled vocabulary is something that the community may wish to agree upon. For example, one question we pose is — what should we name these DNA sequences that encode digital information? Following the practice of genome scientists, we initially called collections of such DNA sequences *libraries*. However, working with such samples caused confusion with our colleagues in a molecular biology laboratory: in a Next Generation Sequencing context, the term *library* is commonly used to describe DNA fragments that have been prepared for DNA sequencing. We now propose to refer to DNA sequences that store digital information as *inDNA* (for ‘ *in*formation-carrying *DNA*’). To refer to *inDNA* prepared for DNA sequencing, we can now unambiguously talk about a *library of inDNA*.

We would like to invite others to contribute to the development of a controlled vocabulary so that we might be able to communicate more precisely. We have included a few entries within our glossary document.

## Improving review, accessibility and impact of interdisciplinary publications

We now pose another question — how might we improve data description and presentation to increase accessibility and facilitate peer review and reproducibility? Peer review is crucial within the scientific community, but this quality improvement process may not be fully realised in interdisciplinary publications. We have experienced difficulties with peer review of publications related to DNA-storage applications, as authors of work under review, as reviewers ourselves, in our assessment of others’ reviews, and in dealings with journal editors. Often the expertise is not available, or reviewers may only evaluate limited aspects of the paper. The body of work may not be effectively reviewed as a whole, leaving authors without vital feedback and potentially leading to publication of flawed work.

### Presentation can be improved by including a short plain-language summary

The concept of standardising presentation of data and methods is not a novel idea in the life sciences, with ‘minimum information’ standards ensuring that publications contain the information necessary to interpret the experimental data. These are typically technique- or study-specific, e.g. MIAME (microarray experiments) ^12^, MIQE (quantitative polymerase chain reaction) ^13^ and MIFlowCyt (flow cytometry) ^14^. Such an approach may not be appropriate to publications relating to DNA-storage applications for some time, as these typically encompass a number of disciplines, each with its own established data description standards and many of which use rapidly changing technologies. It is not appropriate or practical to standardise such a diverse range of technologies and disciplines. Rather we should respect the accepted discipline norms, blending these together to permit DNA-storage standards to evolve.

Even publications that sit predominantly within a single discipline may be of interest to those unfamiliar with that discipline and benefit from the inclusion of a whole-paper plain-language summary. As standard with plain-language summaries this should simply report the basic rational, methodology and main findings. Box 1 is a whole-publication plain-language summary of 2 that we have written as an example.

Box 1. **Plain-language summary of 2.**

With the amount of digital information that needs to be stored growing exponentially there is a need to develop new ways of storing information. High information capacity, longevity and constant improvements in technologies that allow writing, copying and reading make DNA an attractive medium for storing digital information. Here we present a scalable reliable method for storing digital information in DNA.

The original bytes of several computer files in various formats were encoded into DNA as follows. A Huffman code was used to compress each byte, depending upon occurrence frequency, into a block of 5–6 trits, which are the characters 0, 1 or 2 (just as bits are 0 or 1). A reference table of these blocks and corresponding nucleotide sequences was created, with each block having four possible nucleotide combination representations. Nucleotide combinations were selected depending also upon the previous block, in a manner that prevented the occurrence of any repeating nucleotides (e.g. AA), as these are known to cause downstream copying and reading problems. Following encoding the digital information was represented as 153,335 DNA sequences of length 117 nucleotides, each containing an index and a simple error checkpoint in addition to encoding part of the original digital information. These DNA sequences were printed as a pool of DNA, containing ~1.2 × 10 ^7^ copies of each sequence, which was copied via PCR and prepared for reading via DNA sequencing before being decoded (encoding strategy reversed).

Data totalling 739 kilobytes was successfully encoded into DNA, printed, copied, read and decoded with 100% accuracy. A storage density of ~2.2PB g ^−1^ DNA was achieved.

**Figure 1.**
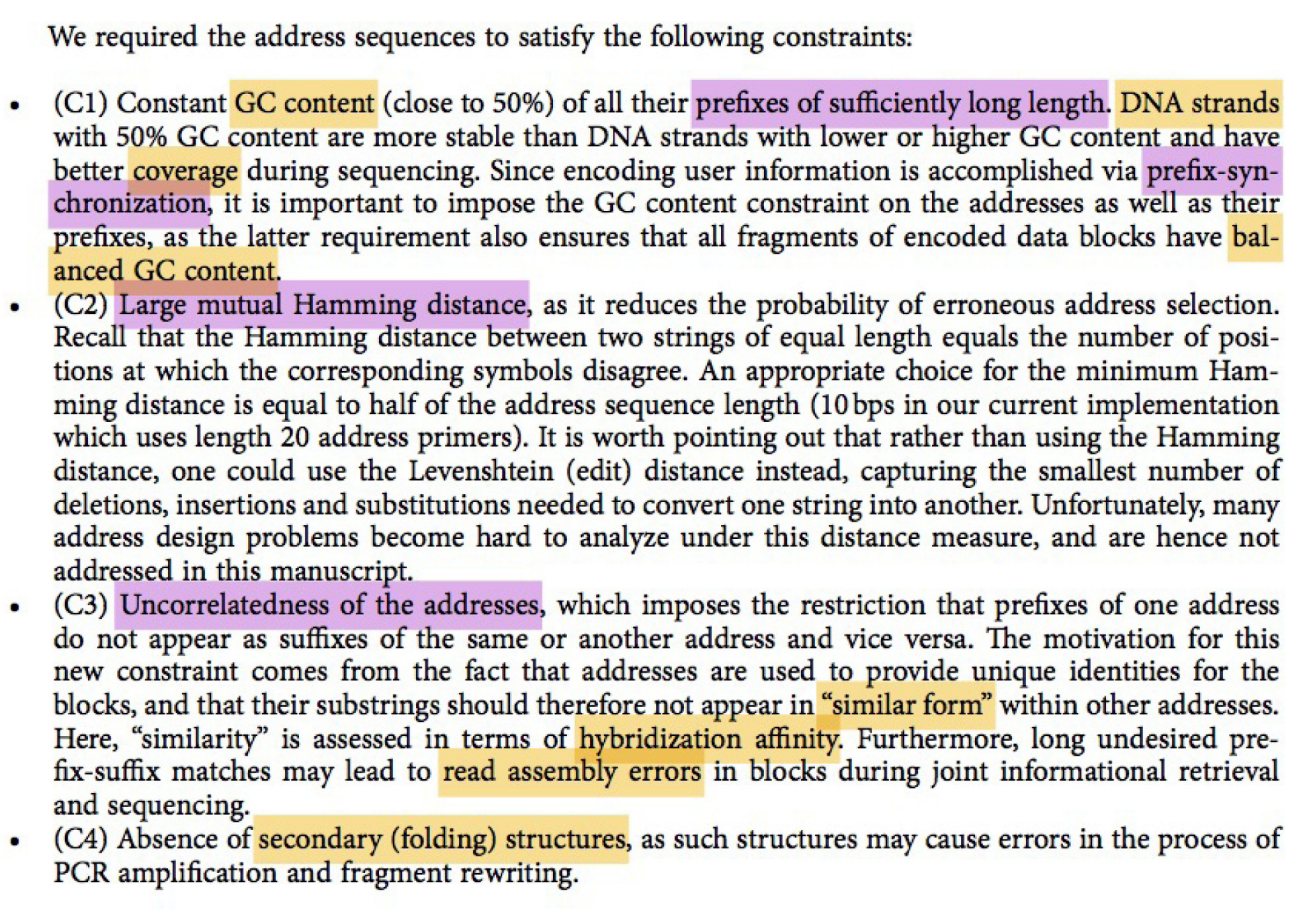
Sample paragraphs from 4. Terms that may not be clear to non-specialists in particular fields are highlighted in purple and yellow, corresponding to those causing problems for a molecular biologist and an information theorist, respectively. (Used under the Creative Commons Attribution 4.0 International License: http://creativecommons.org/licenses/by/4.0).

It may also be useful to provide a plain-language summary of a specific technical aspect of a publication. For example, a molecular scientist may not understand the details of a complex mathematical algorithm (and nor should the description be altered specifically to allow them to), but an appreciation of how the output impacts aspects of the project relevant to them may be sufficient. We illustrate this using a paragraph from 4 (from p.5, *Methods — Address Design and Encoding*). This was read and discussed by the first two co-authors of the present paper, EEH and JS. Figure 1 highlights terms that either EEH, a molecular biologist (purple shading), or JS, an information theorist (yellow shading), found difficult to understand. Joining forces and explaining all terms to each other, they were able to understand the paragraph in depth.

As the interdisciplinary field of DNA-storage evolves towards maturity, there will be an increasing requirement for researchers from different backgrounds to understand publications without having access to colleagues from unfamiliar subject areas. This can be achieved in part by including brief summaries, which may make use of our glossary document, in specialised sections of a publications such that they become accessible for researchers from all disciplines.

## Conclusions

We promote the value of interdisciplinary, collaborative science to solve complex problems, including in our field of digital information storage in DNA which combines molecular biology, information theory and computer science. We note the problems that this approach can generate in communication within and between research teams, and propose to reduce these in the DNA-storage area by initiating a glossary and controlled vocabulary. These have been made available to the research community for reference and critique, and we invite contributions to extend their scope.
