# Supplementary material for: Improving communication for interdisciplinary teams working on storage of digital information in DNA
Source: F1000Res. 2018 Jan 10;7:39. [Version 1] doi: 10.12688/f1000research.13482.1 (PMC5883387; doi:10.12688/f1000research.13482.1)
Supplement: Peer review history

# Peer review: Referee response for version 1

**DOI:** 10.5256/f1000research.14640.r31971

**Published:** 2018-03-29

**Authors:** Jeffrey R. Sampson

**Affiliations:** 1 Agilent Research Laboratories, Santa Clara, CA, USA

The paper by Hesketh *et al.*, addresses the very important issue of facilitating productive communication among highly interdisciplinary teams. This impacts not only verbal communication among interdisciplinary members but also written communications in the form of simple messages and publications.  It is also well noted that during peer review of publications, there is often lacking a single person with the necessary vocabulary and domain knowledge to fully understand, evaluate and communicate a review of the work. The method of Hesketh *et al*. will clearly aid in this important process. Importantly, they have developed a smart approach to the problem that can be applied more broadly to other interdisciplinary teams that require the integration of disparate fields of science and technology such as life sciences and engineering. For example, the synthetic biology community has experienced this issue as it has developed and evolved over the past 15 or so years.

 More specifically, Hesketh *et al*. not only set a good structure and context that the interdisciplinary team developing the DNA as a digital information storage media face, but also provides some solutions to critical problems.  The first is creating a glossary of terms so that all disciplines involved can communicate with a common and known set of terms. Second, they have put forward the use of a “controlled vocabulary” where terms that are particular to the emerging interdisciplinary field are defined so as to enable all members to communicate precisely and thus reduce confusion that often occurs when terms have multiple meanings and/or field dependent meanings.  Perhaps most importantly, Hesketh *et al.*, have built their approach as a “living document” where the vocabulary and common vocabulary can be continuously updated by the interdisciplinary community as the community grows and evolves. 

 With respect to any additional comments or edits, I offer that the authors consider adding “Chemistry Terminology” to their glossary with specific attention to the chemical synthesis of DNA since this is the current method for DNA synthesis. Such terms could include; phosphoramidite, cycle yield, coupling efficiency, de-block step, oxidation step.

 Given the importance, clarity and potential for broad applicability, I strongly recommend the paper for indexing.

I have read this submission. I believe that I have an appropriate level of expertise to confirm that it is of an acceptable scientific standard.

# Peer review: Referee response for version 1

**DOI:** 10.5256/f1000research.14640.r29657

**Published:** 2018-02-26

**Authors:** Robert Grass

**Affiliations:** 1 Institute for Chemical and Bioengineering (ICB), Department of Chemistry and Applied Biosciences (D-CHAB), ETH Zurich (Swiss Federal Institute of Technology in Zurich), Zürich, Switzerland

The paper by E. Hesketh addresses very important problems of our current scientific landscape, and the ongoing movement to more interdisciplinary approaches:

- Communication between scientists in a team
- Peer Review

 

 The authors discuss these two topics using a currently evolving research topic: the storage of digital information in DNA; but the addressed problems have a significantly broader applicability, as individual research topics spread over more and more scientific disciplines, and especially because data and computer sciences are having a major impact on science (and the corresponding high-level mathematics are currently not integrated into e.g. life-science curricula).

 For the communication for scientists within a team, the authors present an excellent glossary of terms for the scientific fields involved in DNA data storage - and the development, and open publication/distribution of such glossaries would bring benefit to many interdisciplinary projects. Instead of a locally managed glossary (as proposed), are more open approach (e.g. as an open Wikipedia) would be even more beneficial and further motivate others to participate stronger in updating the glossary. Additionally, some referencing within the glossary would be additionally valuable - as often background in understanding an individual term is required. (as standard within Wikipedia). If the authors have good reasons for a non-public (i.e. wiki) approach, theses should be discussed in the article, if not, the generation of a corresponding wiki would be certainly highly appreciated by the research community.

 However, to completely solve the communication problems and misunderstandings in such projects, the authors touch a point of even higher importance: “misunderstandings can pass unnoticed”, so the question is what solutions are available to make team members aware of the danger of miscommunication and, implement sufficient effort for every individual in a given project to learn the details, wordings and backgrounds of the neighboring fields- the authors may want to further build on this observation, and potentially present approaches to ensure such awareness and openness (especially in teams involving specialists).

 The second problem of interdisciplinary projects addressed is peer-review. The more detailed background of different scientific fields is required to judge the correctness of scientific work performed, the more difficult it is to find individuals as paper referees who cover all of this knowledge. A plain text summary, as presented by the authors as part of a solution is certainly a good start, but probably does not go far enough. In contrast to individuals working on an interdisciplinary project (as above), a journal referee does not have enough time to learn details and wordings of the other fields, and the review process gets somewhat superficial. A general understanding of the overall goals of a given paper (as per plain text summary) may help the referee to understand the article scope, but it will not help him to judge the scientific validity of the methods applied. The authors of the present manuscript somewhat touch on this, and a more explicit depiction of the problem may be valuable to a further discussion of future publishing/peer-review modes (e.g. post-publication review, open-review, various referees only refereeing part of articles).

I have read this submission. I believe that I have an appropriate level of expertise to confirm that it is of an acceptable scientific standard.
